# IoT Sensor Challenges for Geothermal Energy Installations Monitoring: A Survey

**DOI:** 10.3390/s23125577

**Published:** 2023-06-14

**Authors:** Michal Prauzek, Tereza Kucova, Jaromir Konecny, Monika Adamikova, Karolina Gaiova, Miroslav Mikus, Pavel Pospisil, Darius Andriukaitis, Mindaugas Zilys, Birgitta Martinkauppi, Jiri Koziorek

**Affiliations:** 1 Department of Cybernetics and Biomedical Engineering, VSB—Technical University of Ostrava, 708 00 Ostrava, Czech Republic; tereza.kucova@vsb.cz (T.K.); jaromir.konecny@vsb.cz (J.K.); monika.adamikova@vsb.cz (M.A.); karolina.gaiova@vsb.cz (K.G.); miroslav.mikus@vsb.cz (M.M.); jiri.koziorek@vsb.cz (J.K.); 2 Department of Geotechnics and Underground Engineering, VSB—Technical University of Ostrava, 708 00 Ostrava, Czech Republic; pavel.pospisil@vsb.cz; 3 Department of Electronics Engineering, Kaunas University of Technology, 44249 Kaunas, Lithuania; darius.andriukaitis@ktu.lt (D.A.); mindaugas.zilys@ktu.lt (M.Z.); 4 School of Technology and Innovations, University of Vaasa, 65200 Vaasa, Finland; birgitta.martinkauppi@uwasa.fi

**Keywords:** geothermal energy, geothermal sensors, energy harvesting, edge computing, IoT

## Abstract

Geothermal energy installations are becoming increasingly common in new city developments and renovations. With a broad range of technological applications and improvements in this field, the demand for suitable monitoring technologies and control processes for geothermal energy installations is also growing. This article identifies opportunities for the future development and deployment of IoT sensors applied to geothermal energy installations. The first part of the survey describes the technologies and applications of various sensor types. Sensors that monitor temperature, flow rate and other mechanical parameters are presented with a technological background and their potential applications. The second part of the article surveys Internet-of-Things (IoT), communication technology and cloud solutions applicable to geothermal energy monitoring, with a focus on IoT node designs, data transmission technologies and cloud services. Energy harvesting technologies and edge computing methods are also reviewed. The survey concludes with a discussion of research challenges and an outline of new areas of application for monitoring geothermal installations and innovating technologies to produce IoT sensor solutions.

## 1. Introduction

Geothermal energy is characterized by two types, one with high enthalpy and the other with low enthalpy. High-enthalpy energy is found in specific regions and rock masses that contain high temperatures and high heat potential, especially volcanic areas and deep faults. High-enthalpy energy is used for power and heat generation, while low-enthalpy energy is used only for heat generation and can be found in most shallow rock mass zones around the world. Underground geothermal structures have been used for various applications, for example, electricity production or district heating. Heat pumps for geothermal applications and geothermal plants produce both electricity and hot water for district heating. To provide the data required for the proper functioning and control of systems, it is necessary to monitor various parameters within specific volumes of rock [1]. Monitoring the conditions of the rock environment provides the information required to manage and control processes to prolong the lifetimes of systems and avoid outages in operating underground geothermal structures [2]. To control a geothermal system, the key monitoring parameters are the fluid temperature, ambient rock environment temperature, volumetric flow rate, and physical characteristics of underground structures; minor monitoring parameters include scale formation [3] and slope movement [4]. Monitoring the specific characteristics and behaviors of underground structures assists in detecting problems and failures that prevent optimal system operation and energy management. The monitoring focus for an underground geothermal structure is dependent on the geothermal application area, examples of which include heat pumps [5], mines or wells [6], pipelines [7], reservoirs [8] and borehole exchangers [9].

Monitoring aims to determine or continuously update the status of key parameters necessary for the proper operation of an underground geothermal structure. To monitor these parameters, devices such as those connected to the Internet of Things (IoT) can be used. These devices must be able to monitor several variables and wirelessly transmit measured values to the cloud [10].

IoT technology is widely used and can also be applied to many areas of monitoring underground geothermal structures. This type of technology is especially useful in locations where wireless communications are required. These areas are generally difficult or impossible to connect to a power grid, and therefore, alternative power sources such as batteries or energy harvesting methods are necessary [11]. In this case, monitoring devices must consume low amounts of energy, and suitable technologies with powering constraints, for example, low-power wide-area network (LPWAN) technology, are therefore required. LPWAN technology was designed for reliable wireless data transmission with minimal power consumption. The commonly used LPWAN technologies include LoRaWAN, Sigfox and NB-IoT [12].

Table 1 lists recently published reviews of monitoring performed in specific underground geothermal structures. The listed studies examined a variety of systems that monitor different parameters, are deployed in specific types of geothermal structures and employ IoT technology. In Refs. [13,14,15], the authors discussed temperature monitoring and the temperature measurement methods, sensors and tools suitable for geothermal applications, such as vertical borehole heat exchangers, downholes and power plant devices. In Refs. [1,2], the authors discussed flow rate sensing techniques, providing comprehensive descriptions and comparisons of the two-phase flow monitoring methods applied in geothermal pipelines and geothermal wells. In addition to monitoring temperature and flow parameters, geothermal applications also use sensors to monitor mechanical parameters, such as scale formation [3] and slope movement [4]. In Ref. [4], the authors provided an overview of wireless sensor networks (WSNs) combined with IoT devices for monitoring slopes. The monitoring of other mechanical parameters has not yet been summarized in any recently published survey. The authors of [7,9] discussed the use of WSNs in underground coal mine monitoring systems and methods for monitoring leakage and sinkholes. The motivation for the current review is the lack of comprehensive studies on IoT applications for monitoring underground geothermal structures. To our best knowledge, no reviews have summarized the use of IoT technology in relation to monitoring geothermal parameters, such as temperature, pressure, flow rate and mechanical parameters.

The main contributions of this article are:

- A comprehensive analysis of methods for monitoring specific parameters in underground geothermal structures and the currently available monitoring technologies and sensors;
- A description of the state-of-the-art IoT technologies and sensors suitable for deployment in underground geothermal structures, including sensor node designs, energy harvesting methods, communication interfaces and cloud services;
- Proposals for new, promising application areas and innovations in technology for monitoring underground geothermal structures.

**Figure 1:**
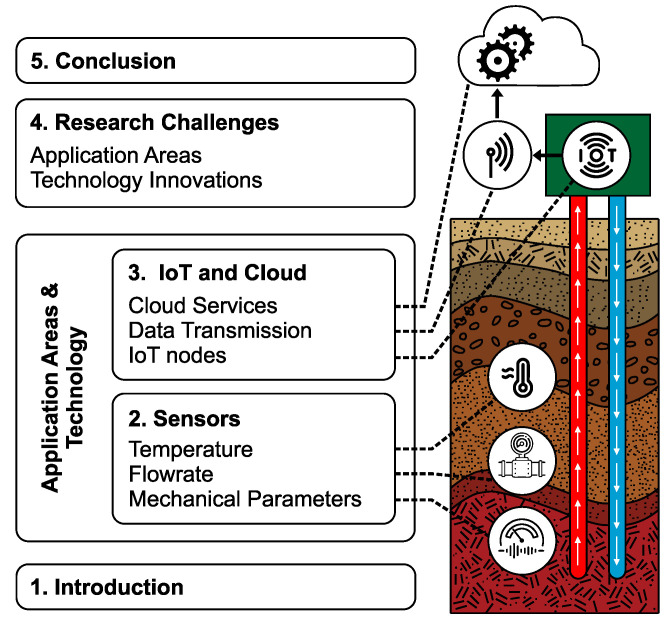
Scheme of the article: sensing parameters in underground structures, IoT technology and cloud processing.

The scheme of the article is illustrated in Figure 1. The article discusses sensing methods for detecting temperature, flow rate and other mechanical parameters in underground structures. Section 2 of the article provides a deeper analysis of the methods applied to monitor these parameters. Section 3 discusses IoT technology, data transmission and cloud services in relation to the systems deployed to monitor underground geothermal structures. Section 4 discusses research challenges. Section 5 summarizes and concludes the article.

## 2. Sensor Technologies

Sensors containing specific technologies are necessary for monitoring the subterranean environment of a geothermal structure. The ability to continuously monitor such structures at a reasonable cost is desirable in terms of improving efficiency and allowing the immediate detection of problems and faults [2]. This section examines the technologies for measuring temperature, flow rate and other mechanical parameters, such as pressure and slope movement, in these specific environments.

### 2.1. Temperature Measurement

Temperature monitoring technologies enable the study of geological processes and the investigation of subsurface thermal properties. Table 2 lists some of the temperature measurement technologies applied in complex systems. The principles of these specific temperature measurement technologies are further described in this subsection.

Depending on the installation method, temperature sensing technologies fall into two categories: static installations and mobile sensing. Static installation systems are fixed and permanently installed. These systems include optical fibres, distributed temperature sensing (DTS) technology, thermistors and thermocouples. Static installation sensors are used for temperature measurements in geothermal wells, downholes or borehole heat exchangers. Mobile sensing uses wireless technology and is not fixed or permanently installed. Mobile sensing systems are used, for example, to monitor the temperature of geothermal fluid in borehole heat exchangers.

#### 2.1.1. Static Installations

Static installations deploy thermistors, thermocouples, fibre optic thermometers or DTS technology. Thermistors are sensors capable of measuring high-quality temperature–depth information from boreholes up to 100 m or more below the Earth’s surface [19]. Thermocouples are installed to measure temperatures at the inlets and outlets of geothermal heat exchangers [20]. Wired optical sensors provide continuous measurements with high-resolution temperature profiles along pipelines [18] and are often attached to the outer surface of the pipe wall or located within the underground structure itself [21].

DTS technology is used for temperature measurements along various types of underground geothermal structures. Measurements are based on detecting light from Rayleigh, Raman or Brillouin backscattering phenomena [15]. DTS technology measures temperatures using optical fibres that provide a continuous temperature distribution profile of the entire cable [22]. This method is able to achieve highly accurate temperature measurements over long distances, up to 30 km. This technology is very sensitive for monitoring temperature, as it is characterized by a spatial resolution of 1 m at an accuracy of $\pm 1{\;}^{\circ }$C and a resolution of 0.01 ${}^{\circ }$C [23]. This technology works by detecting thermal changes along the optical fibre that manifest as molecular or lattice vibrations in the material. In addition, thermal changes cause local changes in the refractive index. Changes in the refractive index are then manifested as the inelastic scattering of transmitted light. Molecular vibrations at high frequencies around 10 THz cause Raman scattering. Brillouin scattering is caused by low-frequency vibrations in the range of 10 to 30 GHz. This scattering can then be used to measure the temperature along the fibre [24].

DTS technology is widely applied to inspect subsurface thermal properties. DTS can be used in various applications, such as investigating the hydro-geological conditions of downholes or determining the geological properties of aquifers. Especially in deep boreholes, its application provides notable benefits over discrete-point temperature measurements [5]. DTS is widely used in long-term monitoring to determine the contribution of groundwater flow and thermophysical characteristics to subsurface heat transfer and storage [16]. This type of technology is used to monitor the conditions inside well bores to optimize expensive workover procedures [17].

#### 2.1.2. Mobile Sensing

Mobile sensing systems such as Geoball and Geosniff are small spheres 25 mm in diameter and 8 g in weight that have an embedded transceiver, microcontroller, temperature sensor and power supply. These miniaturized sensors are inserted into the pipes of a geothermal underground structure and are then carried by the thermal fluid in order to monitor the thermal parameters of the fluid along the borehole heat exchanger. These sensor systems allow the acquisition of information on the thermal properties of the subsurface geological structure and their influence on the thermal properties of the borehole. A wireless data processing unit sends data and acquires configurational information prior to measurement and retrieves measured thermal data from a sensor inside the U-tube of the borehole heat exchanger [18].

These types of sensors have demonstrable advantages of higher reliability and lower cost over standard sensors and fibre optics [13]. Fibre optic technologies such as high-temperature distributed acoustic sensors [25] are used for their unparalleled ability to discriminate and measure environmental variables such as strain, temperature and vibrations [26]. The purpose of the method is to improve the conventional thermal response test [18]. Compared to Pt100 sensors, mobile sensing systems are highly accurate and do not produce large, statistically significant measurement deviations [13].

### 2.2. Flow Rate Measurement

The measurement of liquid and gaseous flow rates in underground geothermal structures is performed to manage and control various processes [1]. Flow rate measurement in geothermal structures applies techniques that differ in complexity, cost and the capability to perform real-time surveys. Table 3 summarizes the differences between individual methods of flow rate measurement on the basis of four attributes: the ability to provide real-time measurements (real-time), the operational status of the underground structure (out of operation), direct contact between the medium and the sensing device (direct contact) and a list of the types of underground structures where the flow rate measurement method is used (application location).

In summary, each method, except for the calorimeter and tracer dilution, provides real-time measurements. The calorimeter and lip pressure are the only two methods used in decommissioned underground structures. Each method, except for compression load cells and the ultrasonic meter, has direct contact with the sensing medium. The application location includes geothermal power plants, open Venturi channels, geothermal pipelines and geothermal wells.

Flow rate measurement can apply single flow rate methods or hybrid flow rate methods. Hybrid methods combine two basic methods to reduce any deficiencies that single methods may introduce. Hybrid techniques are frequently used to measure two-phase fluid flow. The following subsections describe the individual methods listed in Table 3 for flow rate measurement and estimation.

#### 2.2.1. Ultrasonic Meter

Ultrasonic meters are devices that are composed of a receiver and transmitter and that use the phenomenon of sonic waves. The device is attached to the exterior of a pipeline and used to determine the flow rate. The transmitted waves are detected by the receiver. The modification of the wave frequency and amplitude is indicative of the properties of the fluid/gas. There exist several methods that can be used to calculate the final flow rate, for example, based on the time of transit of the sonic waves [36], the Doppler Effect [37], cross-correlation [37] or changes in wave amplitude [38]. Ultrasonic sensors are used in many areas of industry. In underground structures, at least three sensors are used at different points [28], for example, at different locations along a channel [27]. The advantage of an ultrasonic meter is that it does not come into contact with the fluid/gas medium and is positioned on the outside of the pipeline [27].

#### 2.2.2. Calorimeter

**Figure 2:**
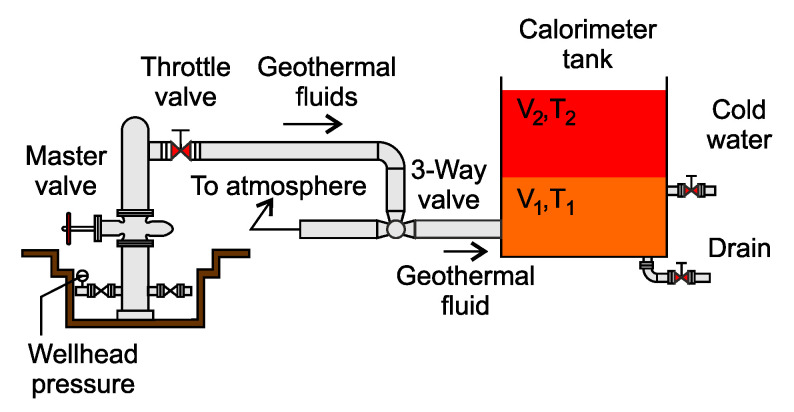
Block diagram of a calorimeter setup.

Figure 2 depicts a typical calorimeter configuration that is used to measure enthalpy in geothermal wells in addition to the flow rate. This method works on the principle of measuring the time that it takes to fill a partially water-filled tank with geothermal fluid. The time is then used to calculate the flow rate [2].

The disadvantage of this technique is that it requires the geothermal well to halt production [1]. Another disadvantage is that it is only suitable for low-output wells, as the amount of discharge is restricted by the volume of the tank that can be delivered to the site. Another constraint is the limit on the maximum measurable flow rate of 10 kg/s [39]. Measuring the flow rate in this manner is also very noisy and cannot be performed in real time [40].

#### 2.2.3. Sharp-Edged Orifice Plate

**Figure 3:**
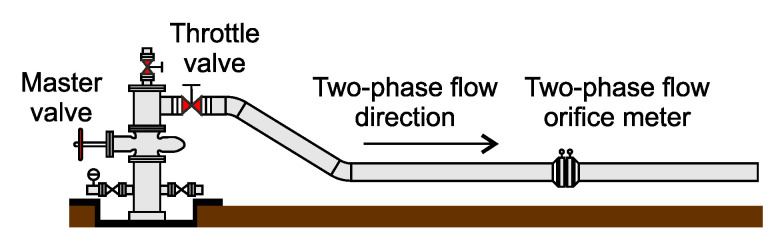
Orifice plate installed at a geothermal well.

An orifice plate is a well-known alternative method that has been implemented in several geothermal applications all over the world [1]. The orifice plate consists of a central circular hole that measures differential pressure by establishing pressure taps upstream and downstream. Figure 3 [2] illustrates a typical orifice plate setup.

Orifice plates are frequently used to measure the flow of geothermal steam; extracting steam from the ground is the first crucial step in converting geothermal steam energy into electricity [41]. The devices also measure brine flow [2] and can identify the mass flow rate of the well [42].

#### 2.2.4. Tracer Dilution Method

Tracer dilution is used to measure the enthalpy of geothermal fluid in addition to the flow rate. To determine the well performance, two different chemical tracers are injected into the fluid stream in the pipe and mixed with water. Fluid tracers disperse into a liquid phase; gas tracers mainly disperse into a gaseous phase, but also partially into a liquid phase. Samples of vapor and water are then collected from the pipeline and tested to identify the dilution of the tracer in both phases and the background concentration. From this information, the flow rate and enthalpy can be estimated [1]. The advantages of this method are the ability to keep the well online during measurement and the lack of an effect on the flow; they are outweighed, however, by the large costs for lab testing [1], the poor precision of the results [2] and the lack of real-time measurement capability [43].

#### 2.2.5. Lip Pressure Method

This method entails the use of a small hole measuring 6 mm in diameter and located 6 mm away from the pipe lip. Empirical measurements of pressure are taken at this hole, and using the size of the pipe, an empirical correlation is used to calculate the flow rate. Enthalpy and the total flow can be determined based on information obtained from the flow rate and lip pressure [32]. Two arrangements are available for this method, one in which fluid is discharged into the atmosphere through a vertical lip pressure pipe and another in which it is discharged horizontally into a silencer on a horizontal lip pressure pipe. In the vertical arrangement, the pipe is connected to the top valve in a vertical position, and the fluid’s enthalpy must be estimated based on the temperature of the primary supply zone [1]. In the horizontal arrangement, the pipe discharges into the silencer, and the weir box located immediately downstream of the silencer monitors the water flow. The method’s advantages include low running costs and real-time measurements, but it also has drawbacks, such as high noise levels and interruptions in operations during measurement [44].

#### 2.2.6. Venturi Tube

The Venturi tube method is based on Bernoulli’s principle and is a very precise method used for measuring steam flow [34]. The method can be used for online or real-time measurement, and its main advantage is a low and constant pressure drop. Its construction is complicated and expensive, however [2,45].

#### 2.2.7. Coriolis Flow Meter

A Coriolis flow meter consists of two curved tubes that vibrate symmetrically. The flow of the medium is directed through these pipes, and any local changes in the vibration frequency due to the flow rate are monitored. To calculate the flow rate, changes in the frequency are measured by sensors, and empirical correlations are applied. Although this method enables real-time and online measurement, it is highly inaccurate and therefore an unpopular option [2,46].

#### 2.2.8. Compression Load Cells

**Figure 4:**
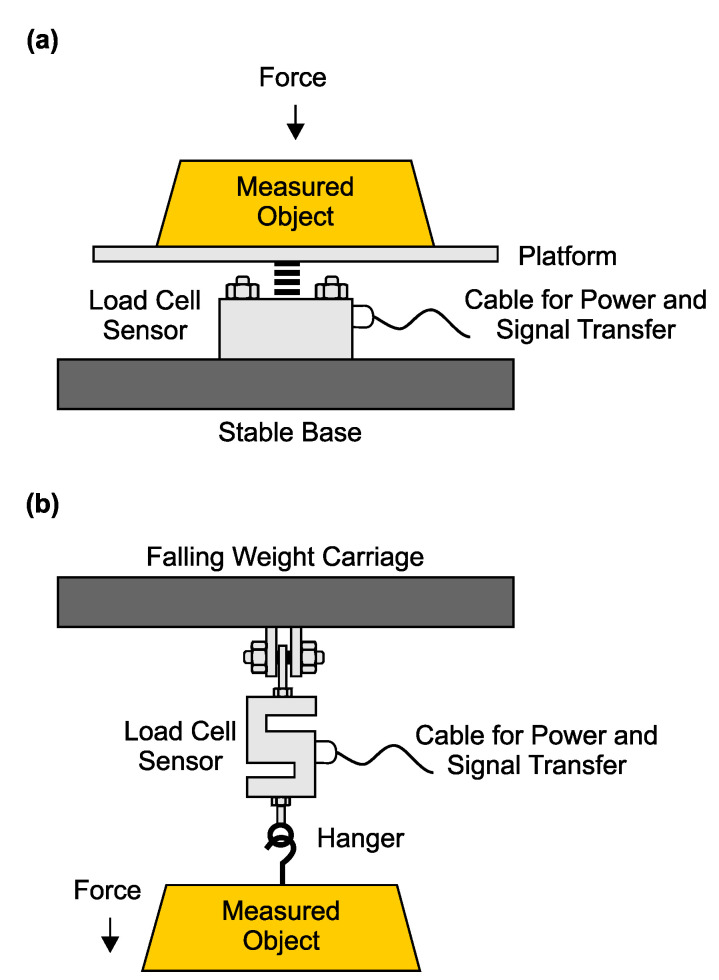
Compression load cell method: An installation in a geothermal pipeline. (**a**) The load sensor is positioned above a platform. (**b**) The load sensor is positioned above an object.

Compression load cells use strain gauge sensors to determine the flow rate and enthalpy in geothermal pipelines and wells. The sensor is based on the piezoelectric effect, which is based on converting the mechanical power applied by the object (pipeline, well) to an electrostatic signal. The load sensor is positioned above a platform (compression type, Figure 4a) or above the object (tension type, Figure 4b) [35,47].

The advantages of this method are simple construction, measurement in real time and the lack of direct contact with the fluid medium [35,47].

#### 2.2.9. Separator with Orifice Plate and Silencer

This hybrid method determines the flow of a two-phase fluid and consists of a separator, an orifice plate and a silencer with a weir. The fluid separates into vapor and a liquid in a separator vessel at a pressure greater than atmospheric. The separate steam flow is measured with an orifice plate, while the separate liquid flow is determined with either an orifice plate or a silencer with a weir box. Because orifice plates may produce unstable measurements due to flash evaporation induced in the liquid, silencers are more commonly used for measuring the separate liquid flow. The separator method is expensive to implement, but it enables high accuracy and online or real-time measurement [1,2].

### 2.3. Measurement of Mechanical Parameters

In addition to measuring temperature and flow, monitoring mechanical parameters in underground structures is important for detecting potential risks. Underground structures such as borehole heat exchangers must be regularly checked for pressure to ensure that no fluid leaks into the subsurface, groundwater or process water. Geothermal applications also use sensors to monitor other mechanical parameters, such as scale formation [3] and slope movement [4]. Mechanical parameters can be monitored with fibre-optic sensors [48], tilt sensors [4], ultrasonic sensors [49], vibrating wire sensors [50] or strain gauges [51]. Table 4 summarizes these sensing methods and their areas of application.

Pressure is measured with strain gauges or optical sensors, usually in geothermal wells or reservoirs. Optical sensors are also used to detect the formation of scale in geothermal brine. In boreholes, ultrasonic sensors are deployed to detect breakouts, and tilt sensors measure information about slope movement and deformation. Vibrating wire sensors are used to measure stress in structures at geothermal or oil drilling wells.

#### 2.3.1. Optical Sensors

Fibre-optic cables are used to measure pressure in geothermal wells [48]. Distributed acoustic sensing systems use fibre-optic cables to measure oscillatory strain rates caused by oscillatory pressure changes. These cables have significant advantages in being able to withstand harsh environments; they can be deployed for geomechanical, seismic and temperature monitoring [53]. Fibre-optic cables are capable of operating at temperatures up to $315{\;}^{\circ }C$ and 20 MPa [51].

#### 2.3.2. Vibrating Wire Sensors

Vibrating wire sensors can measure the stresses occurring in a structure and its surrounding environment. It is a frequency sensor with an internal metal string as the sensing component. The sensor’s output frequency signal changes according to the the stress on the metal string. The device is durable and simple in design and has a strong anti-interference ability [50]. The metal string vibrates as a result of excitation when an external force is applied, changing the tension in the wire, which changes the frequency of vibrations. The dependence between the vibration conductor and the vibration frequency is calculated from the equation:

(1) $$f=\frac{1}{2L}\sqrt{\frac{F}{m}},$$

where *m* is the mass of the vibrating string, *L* is the length of the vibrating string, *F* indicates the pressure on the sensor, and *f* is the resonant frequency of the string [54]. These sensors are suitable for applications in geothermal or oil drilling wells [52].

#### 2.3.3. Tilt Sensors

These sensors are used to obtain information about slope movements and deformations. Tilt monitoring systems combine tilt sensors and LPWAN technology to measure slope movement and transmit collected data wirelessly. Data from tilt sensors and other additional parameters (rainfall data, groundwater level) are analyzed to predict slope failures [55].

## 3. IoT Devices and Cloud Processing

IoT technology is a growing research area. In 2021, the Web of Science^TM^ library contained more than 13,000 indexed papers on the topic of IoT technology [56]. IoT technologies are deployed in many application areas, such as environmental monitoring [10], smart factories [57,58], smart cities [59] and smart grids [11,60]. Although IoT technology is widely deployed, only a few research articles cover the monitoring of geothermal energy installations.

**Figure 5:**
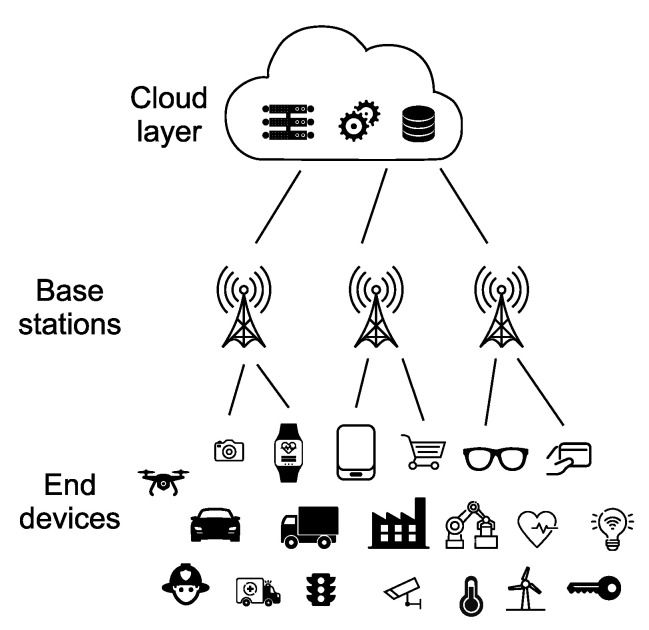
IoT architecture and application domains.

Fundamentally, an IoT device is an internet connection. In the context of this article, an IoT device is a small item of electronic equipment that is able to supervise several variables and transmit measured values to the cloud via a wireless connection. Figure 5 illustrates the principal scheme of the IoT architecture and application areas.

**Figure 6:**
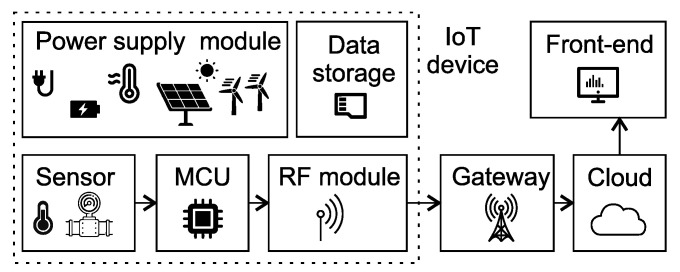
Block diagram of a typical IoT solution.

Figure 6 shows a block diagram of a typical IoT solution. IoT devices generally contain the following parts: a power supply module, a sensory subsystem, a microcontroller (MCU), a transmission module (RF module) and data storage. Each block can be assembled from various components according to system requirements and deployment characteristics.

### 3.1. IoT Nodes

An integral component of an IoT device is a power supply. To supply the device with power, many options are available, the simplest being a connection to a power grid; an example is an AC power monitoring module with an integrated wireless connection via WiFi [61]. Power grids provide sources of stable and reliable energy, but they are mainly only available in urban locations. Another option is the use of a battery [62]. Batteries also provide stable sources of energy, but they require periodic charging and replacement. Battery-powered IoT devices can also be designed to operate for long life spans [10]. Another option for supplying electricity is through energy harvesting methods [63]. Characterized by many advantages, energy harvesting modules are small, maintenance-free and inexpensive solutions, especially compared to battery-powered options. The main drawback of energy harvesting solutions, however, is limited available energy.

Table 5 summarizes IoT power supply technologies according to the energy transducer type. Selecting a suitable power supply type is strongly dependent on the target application [64]. In many geothermal applications [65], power grids are readily available, and generally, no energy harvesting or battery technologies are required. However, IoT measurement devices are often deployed directly in geothermal boreholes, where the installation of a wired power supply is complicated [66]. Energy harvesting is therefore a highly suitable technology for these applications.

Energy harvesting using solar panels or small wind turbines is suitable only for surface deployment, where sunlight and wind are sufficient [72]. For underground structures, thermoelectric generators (TEGs) have significant potential as an energy harvesting technology. TEGs exploit the Seebeck effect to generate energy through temperature differentials [73]. The main challenge with this technology is finding suitable temperature differences or gradients directly in a borehole for exposure to the TEG.

Acting as brains, microcontrollers (MCUs) regulate IoT node behavior, such as sensor data collection, local memory storage and data transmission to the cloud. Since IoT nodes generally have only limited energy for operation [74], MCUs are also responsible for energy management. Naturally, they are selected from ultra-low-power series offered by silicon manufacturers to minimize the MCU’s own consumption. Examples of these series types are STM32L [75], NXP K32L [76] and Microchip PIC32MZ [77]. Specific models are selected according to the application, and generally, higher performance requires more energy. ULP (ultra-low-power) MCUs typically offer multiple switchable modes of operation and the ability to switch off individual peripherals (clock gating) for effective power management. Switching off all components not currently in use thus reduces the overall energy consumption [78,79].

IoT nodes are usually equipped with local data storage, which can be used for either the long-term storage of accumulated values or short-term storage (as a memory buffer). As with other IoT node components, power consumption is one of the crucial parameters in selecting memory and is dependent on the type of memory used. Ferroelectric Random-Access Memory (FRAM) is a type of memory technology with low energy consumption, but its data capacity is limited to a few dozen MB. At the other end of the spectrum of data capacity is flash memory, commonly found in SD cards, with maximum capacities of hundreds of GB. The disadvantage of flash memory is higher energy consumption and significantly slower data write speeds. Data storage memory may form either a discrete component in an IoT node or be integrated into the MCU [80].

### 3.2. Data Transmission Technologies

Underground geothermal structures are often situated in remote areas where no easy connection to the internet for monitoring purposes is possible (i.e., WiFi or Ethernet). In these areas, monitoring equipment is usually powered by batteries or energy obtained by harvesting (see Section 3.1), and energy consumption must be low. GSM/LTE technology does not satisfy this low-power requirement, but LPWAN technology is ideal. These types of systems were developed for the IoT and feature reliable, long-distance wireless data transmission (1–40 km), with minimal power consumption. The commonly used LPWAN technologies are currently LoRa, Sigfox and NB-IoT [81,82].

#### 3.2.1. LoRaWAN

LoRaWAN is a wireless communication protocol based on the patented LoRa technology. The patent was granted in 2014 to Semtec, and the first LoRaWAN protocol specification was released in 2015. The standards are now managed by the LoRa Alliance. Transceivers have a low sensitivity to noise and thus a greater range thanks to forward error correction (FEC), together with a proprietary spread spectrum modulation technique. The operating radio frequency varies according to the region of deployment and its local regulations, but it always belongs to a license-free band. A LoRaWAN network typically includes the following network elements. The end device is application-dependent, provides control or measurement functions (sensor, actuator) and is wirelessly connected to the LoRaWAN network via a gateway. The gateway receives and forwards messages (usually via the internet) to a network server. The network server provides network management functions such as data processing, message deduplication, configuration and scheduling, bandwidth regulation and message routing. The application server remotely collects data from sensors and transmits commands to device actuators [83,84].

The technology has a range of 5 km (urban) to 20 km (rural) [85]. Depending on the configuration, the payload can contain up to 242 bytes of data [86].

LoRa defines three device classes:

- Class A—End devices can start transmission (uplink) at any time, while the base station transmits data only for a short time once the uplink is complete. Each uplink time slot is followed by two downlink slots, after which the receiver switches off. This class has the lowest energy demand.
- Class B—Devices have the same uplink and downlink slots as Class A, plus one extra downlink slot. The end-device receiver is switched on for this extra slot according to a predetermined schedule. The base station is programmed to follow this schedule.
- Class C—Receivers can listen continuously, except in transmit mode. This class of device is ideal for applications that require many downlink transmissions because the base station broadcasts at any time. Its disadvantage is higher power consumption [87].

#### 3.2.2. Sigfox

Sigfox is a company based in France providing network connectivity within the IoT through its eponymous wireless technology. It was founded in 2010 with a focus on low-consumption IoT devices that require the transmission of small amounts of data over longer distances [87].

Sigfox can operate in either simplex (one-way) or half-duplex (two-way) mode. An upload packet can comprise a maximum of 12 bytes of payload, while a download packet is limited to 8 bytes of payload. The operational range of this network reaches up to 40 km [87,88]. Such a value is achieved by combining several techniques. The first of them is the use of ultra-narrowband (UNB); such a narrow frequency selection increases the signal-to-noise ratio (SNR). A combination of Gaussian Frequency-Shift Keying (GFSK) and Differential Binary Phase-Shift Keying (DBPSK) is used for signal modulation in the physical layer. Delivery reliability is increased by sending the same message multiple times. Duplicate messages are sent on randomly selected frequencies with random delays from the first one. Sigfox relies on a license-free band reserved for industrial, scientific and medical (ISM) purposes. The specific frequency then depends on the deployment region. In addition to the maximum transmission power, the regulation also sets a duty cycle limit. Because of this, the end device is limited to 140 messages per day in the case of sending and 4 messages in the case of receiving [88,89].

#### 3.2.3. NB-IoT

Narrowband IoT is another LPWAN technology available today. It was developed by the 3GPP organization, with the first NB-IoT standard defined in 2016. This communication network is based on and is directly linked to the GSM/LTE cellular network standard. Modifications consist of changes in the original technology to best meet the requirements for IoT end-device network connectivity (such as low power consumption, low cost or the transmission of small amounts of data over longer distances). Compared to the LTE protocol, NB-IoT only allows half-duplex at a bandwidth of 180 kHz. Modulation schemes more complex than π/4–QPSK are not supported. Not all parts of the E-UTRA specification are implemented either. Thanks to this aspect, the receiver is simpler and therefore less energy-demanding and lower in price [90,91,92].

The standard allows transmission using both unlicensed bands and licensed GSM/LTE brands in one of the following operation modes:

- Stand-alone operation—When using this mode, NB-IoT is the exclusive user of the selected frequency band.
- Guard-band operation—In this mode, NB-IoT data are transmitted through an unused frequency band next to, or between, LTE bands (the so-called guard band).
- In-band operation—LTE data together with NB-IoT data share the same carrier. This can have a negative impact on the quality of both services [90,93].

The existing LTE network provider often only needs to modify the software to operate the NB-IoT network if it decides to deploy the guard-band or in-band model. This approach is also recommended by the 3GPP organization itself. The usable distance of the IoT node from the base station is up to 10 km [85]. The payload of one message is limited to 1600 bytes. There is no limit on the number of messages transferred per day when using the licensed band. NB-IoT relies, like the LTE protocol, on orthogonal frequency division multiplexing (OFDM) to transmit downlink messages. In the case of uplink transactions, OFDM is replaced by single-carrier frequency division multiple access (SC-FDMA). This reduces the value of the peak-to-average power ratio (PAPR) [94].

#### 3.2.4. Comparison of LPWANs

The information in Table 6 indicates that the individual technologies differ, although they are all specific to LPWANs. The key advantage of Sigfox over its competitors is the size of the area covered by a single base station, up to 40 km in rural areas. Under the same conditions, the main competitors achieve a coverage of approximately 20 km with LoRaWAN and 10 km with NB-IoT [95]. NB-IoT is also limited to areas with LTE networks. In terms of deployment, LoRaWAN allows the creation of private networks, while the other two technologies rely only on the provider deployment model [96]. NB-IoT is aligned with applications that require the transfer of large amounts of data or that need a low-latency network. Sigfox offers a payload volume approximately 100 times lower than NB-IoT (maximum 1600 B). LoRaWAN provides a payload of 242 B, and latency can be reduced using class C devices [86]. All of these technologies offer sleep modes to reduce the energy consumption of a transmitter to a minimum when the end device is not transmitting. NB-IoT nodes have higher power requirements than LoRaWAN and Sigfox, drawing the highest peak currents during transmission through the use of QPSK modulation in combination with OFDM-FDMA and QoS handling [85,97]. LoRaWAN and Sigfox are ALOHA-based and operate on unlicensed frequencies. NB-IoT relies on licensed LTE bands, which ensure higher QoS through regulation, but a consequence is a higher price for the end customer due to license fees [95].

### 3.3. Cloud Services

**Figure 7:**
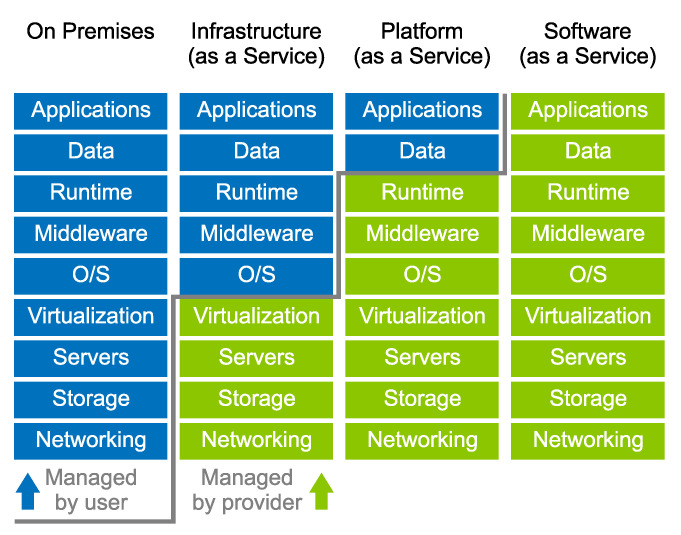
Cloud model types: on premises, infrastructure as a service, platform as a service and software as a service.

Cloud services involve infrastructure, platforms and software delivered on demand to companies and customers via the internet. Cloud services employ cloud computing solutions and do not encumber the hardware or software of devices accessing the service. The variety of available cloud services may disguise the level of an organization’s ownership of the stack [98]. For clarity, cloud service models can be grouped into four main categories according to how they are managed (Figure 7).

- On Premises (Private Cloud)—This cloud model is in fact not a genuine cloud service. It is suitable for users who require data protection and security and are willing to pay more for the service. Because data and all infrastructure, including software and hardware, are on the premises, the system is managed by the user’s own IT team. In-house maintenance requirements and fixed scalability mean that this type of deployment is the most expensive.
- Infrastructure as a Service (IaaS)—IaaS allows customers to outsource equipment such as storage, servers and network resources. The remainder of the stack is managed by the customer. The disadvantage of IaaS is the potential for vendor outages and the cost of training personnel to manage new infrastructure.
- Platform as a Service (PaaS)—The provider manages everything as in IaaS but is also responsible for the base software and operating system, middleware and database. The drawback of PaaS is potential security problems and greater dependency on the vendor.
- Software as a Service (SaaS)—This model completely transfers the management of all components to the vendor service provider [99,100].

Table 7 summarizes the features of three widely used cloud services: Amazon Web Services (AWS), Microsoft Azure IoT, Google Cloud Platform [101,102,103], and IBM cloud [104]. Generally, these state-of-the-art cloud providers dedicate broad support to services and applications designed for the IoT and standard communication interfaces for connecting IoT devices to a cloud. They also feature powerful scalable data storage and support for big data analysis.

### 3.4. Edge Computing

Edge computing is a method with enormous potential for IoT geothermal monitoring. Edge computing allows some computation and data storage to be moved closer to the source of the data, enabling the optimization of IoT node transmissions.

Geothermal installations make use of a specific IoT domain. Table 8 provides a summary of some service-oriented architectures for edge computing methods suitable for use in geothermal installations. Early warning systems can monitor a geothermal installation and evaluate hazardous states. An example of this type of solution is an early warning system for railway bridges [105]. Edge computing also allows the real-time monitoring of natural disasters such as fires [106] or earthquakes [107]. These solutions tolerate multiple node faults and partial network disruptions and store all data locally, which also enhances privacy [107].

Other types of edge computing methods manage data compression. Data compression performed locally significantly reduces the volume of data for transmission [109]. Data compression can be lossy or lossless. The scientific literature on data compression in edge computing is vast [110].

### 3.5. IoT in Geothermal Applications

IoT is chosen over telemetry in geothermal installations due to its ability to provide continuous monitoring and real-time data collection of temperature, pressure, and other mechanical parameters. IoT devices are equipped with sensors that can collect data from various points in the geothermal installation, providing a more comprehensive view of the system’s performance. Additionally, IoT devices can transmit data wirelessly, eliminating the need for wired connections and reducing installation and maintenance costs [111]. Comparison of Telemetry Systems and IoT Device is sumarized in Table 9.

Compared to telemetry, IoT has several advantages in terms of temperature, pressure, or mechanical parameter measurement. Firstly, IoT devices can collect data from multiple sensors simultaneously, providing a more comprehensive view of the system’s performance [112]. Secondly, IoT devices can transmit data wirelessly, eliminating the need for wired connections and reducing installation and maintenance costs [113]. Thirdly, IoT devices can be programmed to send alerts when certain parameters exceed predefined thresholds, allowing for early detection of potential issues and timely intervention.

The lack or improper use of telemetry in geothermal installations can lead to several problems. Firstly, telemetry systems can be expensive to install and maintain, especially in remote locations. Secondly, telemetry systems can be prone to signal interference and data loss, leading to inaccurate or incomplete data. Thirdly, telemetry systems can be difficult to integrate with other monitoring systems, leading to data silos and reduced visibility of the system’s performance [114].

In contrast, IoT devices are relatively inexpensive and easy to install and maintain. Additionally, IoT devices can be integrated with other monitoring systems, providing a more comprehensive view of the system’s performance [115]. Finally, IoT devices can be programmed to send alerts when certain parameters exceed predefined thresholds, allowing for early detection of potential issues and timely intervention.

In summary, IoT is chosen over telemetry in geothermal installations due to its ability to provide continuous monitoring and real-time data collection of temperature, pressure, and other mechanical parameters. IoT devices are equipped with sensors that can collect data from various points in the geothermal installation, providing a more comprehensive view of the system’s performance. Additionally, IoT devices can transmit data wirelessly, eliminating the need for wired connections and reducing installation and maintenance costs. The lack or improper use of telemetry in geothermal installations can lead to several problems, including high costs, signal interference, and data silos [116].

IoT devices have the potential to revolutionize geothermal monitoring, but they also pose several challenges that need to be addressed to ensure reliable operation and minimize the generation of erroneous data. Here are some strategies to confront these challenges:

1. Ensure reliable operation: To ensure reliable operation of IoT devices in geothermal installations, it is essential to choose devices that are designed to withstand the harsh environmental conditions of the installation. This includes selecting devices that are rated for high temperatures, humidity, and corrosion resistance. Additionally, regular maintenance and calibration of the devices can help ensure reliable operation [117].
2. Prevent malfunctions due to exceeding recommended temperature ranges: IoT devices can malfunction if they are exposed to temperatures outside their recommended range. To prevent this, it is essential to monitor the temperature of the devices and the surrounding environment and take appropriate measures to prevent overheating. This can include installing cooling systems or using devices with built-in temperature sensors and automatic shut-off features.
3. Minimize the generation of erroneous data: IoT devices can generate erroneous data due to factors such as sensor drift, signal interference, and data loss. To minimize this, it is essential to use high-quality sensors and ensure that the devices are properly calibrated and maintained [118]. Additionally, data validation and error correction algorithms can be used to identify and correct erroneous data.
4. Transition from elementary sensors to more complex MCU-based IoT devices: The transition from elementary sensors to more complex MCU-based IoT devices can bring several implications, including increased complexity, higher costs, and the need for specialized expertise. To address these challenges, it is essential to carefully evaluate the benefits and drawbacks of using MCU-based IoT devices and ensure that the devices are properly designed, installed, and maintained. Additionally, training and support for personnel responsible for operating and maintaining the devices can help ensure their reliable operation.

In summary, confronting the challenges posed by IoT devices in geothermal installations requires careful consideration of the devices’ design, installation, and maintenance. Strategies such as ensuring reliable operation, preventing malfunctions due to exceeding recommended temperature ranges, minimizing the generation of erroneous data, and carefully evaluating the transition from elementary sensors to more complex MCU-based IoT devices can help ensure the successful implementation of IoT-based geothermal monitoring systems [119].

In the construction and electronic placement of geothermal IoT monitoring systems, careful consideration should be given to the potential high temperatures of an environment or a medium [120]. Geothermal environments can expose the equipment to extreme heat, which can pose challenges to the proper functioning of electronic components. It is crucial to protect the silicon parts used in these systems, as they have maximum temperature limits that should not be exceeded. Adequate measures and safeguards must be implemented to ensure the longevity and reliable operation of IoT devices in such high-temperature conditions [121].

The advance of IoT technology has brought about a push for standardization in various aspects of its implementation. Standardization includes the adoption of common LPWAN communication protocols, the use of standardized components on the cloud side, and the establishment of methodologies for creating IoT nodes [122]. This standardization effort aims to streamline and simplify the development and deployment process of IoT systems. In contrast, telemetry systems often lack standardization and are often proprietary, leading to increased expenses and longer development cycles. The absence of standardized approaches in telemetry results in prolonged development times and delays in bringing products to market, ultimately driving up costs. By embracing standardization in IoT, organizations can benefit from reduced development costs, shorter time to market, and improved interoperability between different IoT devices and systems [123].

## 4. Research Challenges

This article describes options for geothermal monitoring and recent trends in the application of IoT technologies. This section discusses research challenges for potential applications of IoT sensor technology in underground geothermal structures to extend current installations and deliver more robust solutions for future installations. Innovations for specific equipment technologies (sensors, edge-computing, energy harvesting, IoT nodes, etc.) are also discussed.

**Figure 8:**
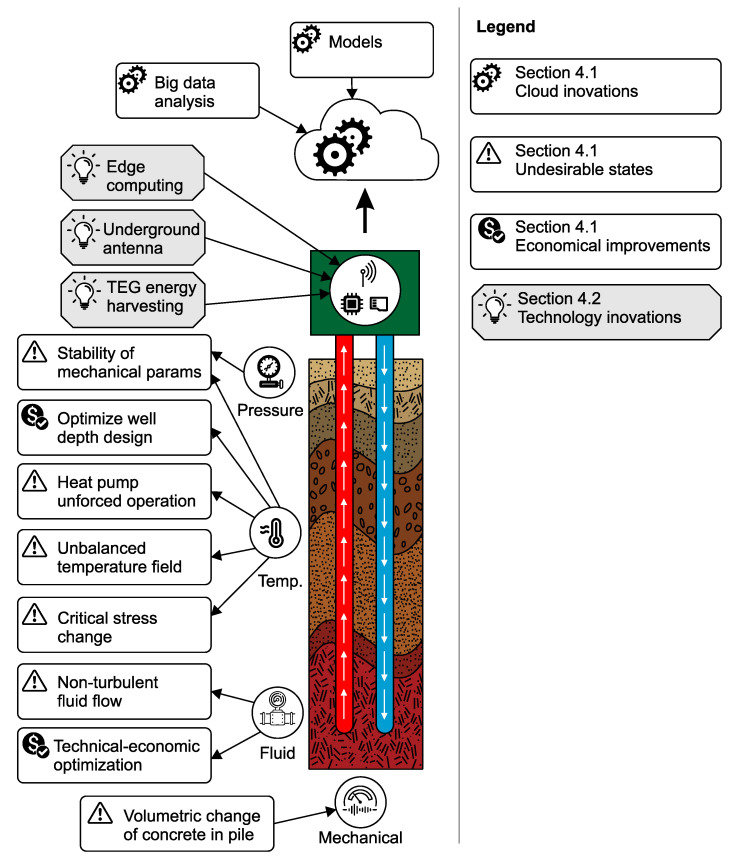
Research challenges for various problematic areas.

Figure 8 illustrates the research challenges outlined in this article, labeled with four different signs. Undesirable states are tagged with a warning sign (exclamation mark within a triangle), innovations for improving the design of future applications are tagged with a dollar sign, innovations for technology are tagged with a light bulb, and innovations for the cloud are tagged with gear wheels. The figure highlights key research challenges and provides ideas for addressing them in the field. Implementation of advanced sensors holds the potential to significantly enhance the design and performance of geothermal installations. The figure introducing the content of the following sections. The Section 4.1 on new potential applications explores emerging uses of technology in innovative ways, opening up exciting possibilities for industries and individuals alike. Technology innovations discussed in the Section 4.2 showcase the latest advancements and breakthroughs that have the potential to revolutionize various fields and create new opportunities for development.

### 4.1. New Potential Applications

Underground geothermal structures are designed for long-term use; therefore, the proper management of the installation’s operational life is required. As mentioned in Section 1, eliminating undesirable states (e.g., thermal imbalance, the absence of turbulent fluid flow and the unforced operation of heat pumps during cooling) during the operation of an underground geothermal structure is beneficial. Long-term monitoring with IoT sensors can facilitate the optimal operation of underground geothermal structures, prolong system endurance and optimize standard operations. Table 10 lists several undesirable states and potential solutions.

Thermal imbalance during the operation of an underground geothermal structure is caused by the heterogeneity of the rock environment. To eliminate thermal imbalances, the monitoring of individual boreholes is a potential solution. The absence of turbulent fluid flow can be avoided with higher flow rates. Low flow rates in the geothermal structure can be specifically addressed with turbocollectors (pipes with spiral ribbing), which guarantee that the Reynolds number of the flow is exceeded. The last undesirable state is the operation of the heat pump during cooling.

The thermal stress of the pile must be monitored for mechanical property stability (strength and deformation parameters). A change in concrete temperature due to heat transfer (charging and discharging) produces a change in the stresses experienced by the concrete in the pile. This change in stress consequently produces volumetric changes in the concrete. In critical cases, the brittle failure of the concrete occurs, and the bearing capacity of the pile as a whole is reduced.

Another benefit of the long-term monitoring of currently deployed geothermal installations is the continuous data collection of operational parameters. Large datasets describing geothermal installation behavior during regular operation and also non-standard conditions can be created. IoT technologies allow the extensive monitoring of existing structures, and therefore, the knowledge base will contain new and important information that is useful for design work for future underground geothermal structures. Table 11 lists parameters whose monitoring could aid in producing improved designs for future applications.

Utilizing measurement and monitoring techniques for borehole properties, particularly temperature and flow rate, presents an opportunity to enhance future applications. Flow rate monitoring could aid in techno-economic optimization through the selection of suitable pipe diameters and optimal energy consumption models. Temperature monitoring could be applied to optimize designs for the well depth (the length of the borehole).

Many underground geothermal structure walls do not permit the use of sensor technologies due to wired sensor limitations or the absence of power grid connections. Such installations may include sensors that are installed during the construction process and are subsequently inaccessible or insular systems where a power supply connection is difficult to build. Energy harvesting technologies have excellent potential as solutions for such cases through their ability to generate electricity for sensor operation directly from the ambient environment.

IoT monitoring systems for geothermal structures can be powered by harvesting energy from the environment. Table 12 lists energy harvesting methods suitable for powering these types of IoT sensors. The most promising energy harvesting method is the use of TEG technology, whose main advantage is the absence of moving mechanical parts and the possibility of placing the device directly into a borehole. Another option is to use water pipeline energy harvesting. This type of energy harvesting system can also be placed into a borehole, but only inside a pipe. Water pipeline energy harvesting modules also have the disadvantage of moving mechanical parts. The third energy harvesting option is solar and wind energy. Solar panels or wind turbines cannot be placed into boreholes, however, and must be located on the surface and be connected to the IoT sensor inside the borehole with wires.

### 4.2. Technological Innovations

The process of constructing an underground geothermal structure requires many technological components. Most of these components do not support direct monitoring, and deploying sensors in existing installations is therefore problematic. The next generation of components could integrate physical sensors directly into equipment. A solution such as this not only permits IoT monitoring but also provides a sensory interface for any type of analytical equipment, such as DTS technology. In principle, components with embedded sensors add value at minimal cost. Technological improvements would involve the installation of optical fibres directly into the underground geothermal structure and the positioning of temperature sensors in the borehole at various depths. These improvements have the potential benefit of optimizing the efficiency of the installation. While this solution addresses certain aspects of the problem, such as improving functionality or performance, it does not still provide a comprehensive solution for maintenance or replacement requirements. In order to ensure sustained effectiveness, it is crucial to consider the high reliability of components involved. Moreover, a key expectation for this solution is its ability to support long-term deployment without significant interruptions or failures.

Energy harvesting transducers can power IoT sensors, but the transducer design must be optimized and aligned with the installation to establish the best energy gains. Thermoelectric generators are a promising technology, but they must be deployed in environments with effective temperature gradients. Temperature gradients for thermoelectric generator purposes are found in environments that fluctuate between medium and ambient temperatures, between warm and cold branches, and between shallow soil (50 cm below ground) and ambient air.

Underground monitoring generally faces problems with data transmission. Regular connections with the cloud can be solved in two ways: a transmission antenna can be placed on the surface, or new types of underground antennae suitable for IoT communications must be developed.

The final research challenge involves innovating the communication interface and cloud data processing. Computationally limited, low-power IoT nodes require effective data transmission solutions that can be delivered through edge computing techniques or event-based approaches (Table 13).

Edge computing allows metadata analysis in situ and provides significant benefits for data transmission; for example, IoT nodes monitoring a geothermal energy installation conserve energy and resources by transmitting data only if a hazardous state arises. The data transmission time and the volume of data stored in the cloud are reduced by storing raw sensor data locally, and the use of cloud computational resources is minimized.

IoT monitoring systems designed for the real-time collection of data from geothermal energy installations can also be optimized with edge computing data compression methods to reduce the total volume of transmitted data. Truncated data can also be uploaded when energy for transmission is limited, followed by detailed data transmission in the future when energy is sufficient.

## 5. Conclusions

This article identifies potential research directions for new applications in geothermal energy installations and innovations for IoT monitoring technology and cloud services. The article contributes an overview of state-of-the-art principles in the design of monitoring systems for geothermal energy installations and a review of the sensor technologies available for use with IoT devices and cloud data processing. Suitable sensor equipment includes devices for monitoring temperature, flow rates and other mechanical parameters. The article also reviews IoT technologies, sensor node composition, options for data transmission and the features of cloud services.

Future research targets and several possible directions are outlined in the Research Challenges section. State-of-the-art solutions provide the potential for new applications that address several problematic conditions in geothermal energy installations. Monitoring systems allow the collection of important datasets that can be used to improve future designs of geothermal energy installations. Several technological innovation techniques could also improve the operation of IoT monitoring devices. Operating nodes in monitoring systems can be powered from harvested ambient energy to prolong the operating time and reduce costs. Edge computing methods can optimize data transmission and power consumption and provide early warnings of faulty or inefficient operating conditions in geothermal energy installations.

## Figures and Tables

**Table 1:** Overview of surveys related to monitoring underground geothermal structures.

Author, Source	Monitored Parameters	Geothermal Structure	Article Content	IoT
Aranzabal et al. [13]	Temperature	Vertical borehole heat exchanger	Comparison of methods	No
Sanjuan et al. [14]	Temperature and pH measurements	Downhole	Sensors and equipment dedicated to geothermal applications	No
Ukil et al. [15]	Temperature	Power plant device	Review of technology and applications	No
Helbig et al. [2]	Two-phase flow	Geothermal pipeline	Comparison of methods	No
Mubarok et al. [1]	Two-phase flow	Geothermal well	Comparison of methods	No
Yadav et al. [4]	Slope	Open-pit mine	Review of WSNs with IoT used for monitoring slopes	Yes
Muduli et al. [9]	Temperature, air humidity, gas analysis, etc.	Coal mine	Review of monitoring possibilities for underground coal mines based on WSNs	Yes
Ali et al. [7]	Leakage and sinkhole	Underground pipeline	Review of methods and WSNs for monitoring leakage and sinkholes	Yes

**Table 2:** Overview of technologies applied to temperature measurement in underground geothermal structures.

Author, Source	Geothermal Structure	Methodology
McDaniel et al. [16]	Geothermal exchange borefield	DTS
Reinsch et al. [17]	Geothermal well	DTS
Bense et al. [5]	Downhole	DTS
Martos et al. [18]	Borehole heat exchanger	Autonomous wireless sensor
Aranzabal et al. [13]	Borehole heat exchanger	Geoball and GEOsniff

**Table 3:** Summary of methods for measuring flow rate in underground geothermal structures.

Method	Real-Time	Out of Operation	Direct Contact	Application Location
Ultrasonic meter	Yes	No	No	Geothermal power plant [27], open Venturi channel [28]
Calorimeter	No	Yes	Yes	Geothermal well [29]
Sharp-edge orifice plate	Yes	No	Yes	Geothermal well [1,2]
Tracer dilution	No	No	Yes	Geothermal well [30,31]
Lip pressure	Yes	Yes	Yes	Geothermal well [32]
Venturi tube	Yes	No	Yes	Geothermal power plant [33], geothermal pipeline [34]
Coriolis flow meter	Yes	No	Yes	Open Venturi channel [28]
Compression load cells	Yes	No	No	Geothermal pipeline [35], geothermal well [35]
Separator with orifice plate and silencer	Yes	No	Yes	Geothermal well [1]

**Table 4:** Summary of methods for measuring mechanical parameters.

Parameter	Sensor Type	Application
Pressure [51]	Strain gauge	Geothermal well
Pressure [48]	Fibre-optic	Geothermal well and reservoir
Slope movement, deformation [4]	Tilt sensor	Borehole
Scale formation [3]	Fibre-optic	Geothermal brine
Formation of borehole breakouts [49]	Ultrasonic sensor	Borehole
Stress [52]	Vibrating wire sensor	Geothermal or oil drilling well

**Table 5:** SoA of IoT power supply technologies.

Author, Source	Power Supply Type	Application
Lin et al. [61]	AC power grid	AC power monitoring module
Grimsley et al. [10]	Primary battery	Environmental monitoring
Das et al. [67]	Solar panel	Air pollution monitoring
Civerchia et al. [62]	Battery	Industrial monitoring
Wang et al. [68]	Kinetic energy—vibration	Agricultural industry
Tran et al. [69]	Radio frequency	Healthcare, room temperature, tire pressure, implantable devices, environmental monitoring, air quality monitoring
Twaha et al. [70]	Thermoelectric generator	Industrial utilities, transportation tools, military devices, space applications, medical services
Roy et al. [71]	Wind	Environmental monitoring, agricultural applications

**Table 6:** Summary of LPWAN parameters [85,95,97].

	LoRaWAN	Sigfox	NB-IoT
Range	5–20 km	10–40 km	1–10 km
Band	Unlicensed ISM	Unlicensed ISM	Licensed LTE
MAC layer	ALOHA-based	ALOHA-based	LTE-based
Payload	242 B [86]	12 B (UL), 8 B (DL)	1600 B
Authentication and encryption	AES-128	No	LTE encryption
Adaptive data rate	Yes	No	No
Modulation	LoRa (CSS)	DBPSK, GFSK	QPSK
Transmission speed	50 kbps	100 bps	200 kbps
Deployment model	Provider, private	Provider	Provider
Adaptive data rate	Yes	No	No
Topology	Star, mesh, P2P	Star	Star
Standardization	LoRa Alliance	Sigfox company with ETSI	3GPP
Power consumption	Low	Low	High
Interference immunity	Very high	Very high	Low

**Table 7:** Summary of parameters and features offered by major cloud providers.

	AWS IoT	IBM	Azure IoT	GCP
Connectivity	MQTT, HTTPS, LoRaWAN	HTTP, MQTT	MQTT, AMQP, HTTPS	MQTT, HTTP
Security	TLS	TLS	TLS	TLS
Data storage	MongoDB, Amazon DynamoDB	IBM cloud object/block/file storage	Azure storage	Google Cloud Storage, Cloud Bigtable, Cloud SQL
Microservices	yes	yes	yes	yes
SDK/Language	C++, Python, JavaScript, Java, Embedded C	.NET, Python, Java, NodeJS	.Net and UWP, Python Java, C, NodeJS	C++, Python, Java, NodeJS, Go

**Table 8:** SoA for edge computing methods.

Author, Source	Application
**Early warning systems**	
Kim et. al. [105]	Real-time scour warning system for railway bridges using remote ambient vibration monitoring.
Dai et. al. [106]	IoT framework for electrical fire monitoring at ancient architectural complex.
Bassetti et. al. [107]	Edge computing early warning system for earthquakes. Local probes detect quakes.
**Data compression**	
Rivet et. al. [108]	Edge computing polynomial regression data compression method for environmental monitoring.
Debski et. al. [109]	Edge computing study of autonomous vehicles and robots concerning with online compression.

**Table 9:** Comparison of Telemetry Systems and IoT Devices.

Factors	Telemetry Systems	IoT Devices
Ease of Installation and Maintenance	Complex, requiring specialized equipment and expertise	Relatively easy, designed for plug-and-play operation
Costs	Expensive, especially in remote locations	Relatively inexpensive, deployable in large numbers
Volume of Information	Limited data on a few parameters	Comprehensive data from multiple sensors
Transfer Rate Requirements	High transfer rate for real-time data transmission	Wireless data transmission, reduced transfer rate requirements
Environmental Conditions	Designed to withstand harsh conditions	Impact from temperature, humidity, and corrosion
Operational Range	Longer range, suitable for monitoring large areas or remote locations	Limited range
Device Replacement	Difficult, especially in remote locations	Easily replaced, plug-and-play operation

**Table 10:** Undesirable states and potential solutions.

Undesirable State	Solution
Thermal imbalance	Monitoring of individual boreholes
Absence of turbulent fluid flow	Higher flow rate or pipes with spiral ribbing (to address low flow rates)
Unforced operation of the heat pump during cooling	Heat pump not operational during cooling
Critical stress change	Temperature monitoring of critical limits with adequate reduction of flow rate
Volumetric change in concrete in pile	Application of pressure cell monitoring at the head of the pile between the pile and concrete slab

**Table 11:** List of parameters whose monitoring could aid in producing improved designs for future applications.

Monitored Parameter	Monitoring Purpose
Flow rate at each borehole	Techno-economic optimization (selection of suitable pipe diameters and energy consumption)
Temperature at each borehole	Optimization of designs for well depth

**Table 12:** List of energy harvesting methods suitable for powering sensors used to monitor underground geothermal structure parameters.

Energy Harvesting Method	Application	Features
Thermoelectric generator	Underground IoT	Fixed to the current technology
Water pipeline energy harvesting	IoT sensors near pipes	Inside pipe
Solar and wind	IoT sensors located on the ground	External technology

**Table 13:** Summary of edge computing techniques.

Edge Computing Technique	Application
Metadata analysis	Early warning systems, hazardous state monitoring
Data compression	IoT monitoring

## Data Availability

Not applicable.
